# Draft Genome Sequence of Heavy Metal-Resistant Soil Bacterium *Serratia marcescens* S2I7, Which Has the Ability To Degrade Polyaromatic Hydrocarbons

**DOI:** 10.1128/genomeA.01338-17

**Published:** 2017-11-30

**Authors:** Rhitu Kotoky, L. Paikhomba Singha, Piyush Pandey

**Affiliations:** Department of Microbiology, Assam University, Silchar, India

## Abstract

*Serratia marcescens* S2I7 is a heavy metal-resistant, polyaromatic hydrocarbon-degrading bacterium isolated from petroleum-contaminated sites. The genome contains one circular chromosome (5,241,555 bp; GC content 60.1%) with 4,533 coding sequences. The draft genome sequence includes specific genetic elements for degradation of hydrocarbons and for heavy metal resistance.

## GENOME ANNOUNCEMENT

The bacterial strain *Serratia marcescens* S2I7 was isolated from petroleum-contaminated soil and grown in minimal medium (Bushnell-Haas broth) amended with the polycyclic aromatic hydrocarbon compound benzo[*a*]pyrene (BaP). This bacterium was found to be highly resistant to cadmium and capable of degrading BaP efficiently. Qualitative and quantitative analysis for the degradation of BaP was done by high-pressure liquid chromatography (HPLC) and gas chromatography-mass spectroscopy (GC-MS) analysis. The isolate *S. marcescens* S2I7 could degrade 80.4% of the total BaP concentration after 21 days of incubation. Whole-genome shotgun sequencing of the *S. marcescens* S2I7 genome was done using one Illumina paired-end library with an average insert size of ~400 bp. The paired-end sequencing libraries were prepared using an Illumina TruSeq Nano DNA library prep kit. The DNA was fragmented by Covaris M220, generating the double-stranded DNA fragment with 3′ or 5′ overhang. The fragments were then subjected to end repair, followed by adapter ligation to the fragments. The products were then PCR amplified with the index primer, as described in the kit protocol, and sequenced using the NextSeq 500 platform. The NextSeq 500 paired-end sequencing run generated ~1 Gb of raw reads.

The sequenced raw data were processed to obtain high-quality clean reads using Trimmomatic version 0.35 to remove adapter sequences, ambiguous reads, and low-quality sequences. These reads were trimmed using a quality score threshold of 20 and a length cutoff of 20 bp. Reference-guided assembly of the sample was performed using SAMtools (1). The procedure for genome annotation was done with the Rapid Annotations using Subsystems Technology (RAST) server and the NCBI Prokaryotic Genome Annotation Pipeline (PGAP) (https://www.ncbi.nlm.nih.gov/genome/annotation_prok) (2, 3). The rRNAs and tRNAs were predicted and annotated using RNAmmer (4) and tRNAscan-SE (5), respectively. The genome of *S. marcescens* S2I7 consists of one circular chromosome (5,241,555 bp; 60.1% GC content). Genome coverage was 94.03%.

The annotation of the genome includes 4,533 protein-coding genes, 81 tRNAs, 22 rRNAs, and 47 pseudogenes. The predicted coding sequences were translated, compared, and searched against the NCBI nonredundant (NR) protein, Clusters of Orthologous Groups (COG), and KEGG databases.

### Accession number(s).

The whole-genome shotgun sequence of *S. marcescens* strain S2I7 has been deposited in GenBank under the accession no. CP021984.
